# Insecticide resistance in dengue vectors from hotspots in Selangor, Malaysia

**DOI:** 10.1371/journal.pntd.0009205

**Published:** 2021-03-23

**Authors:** Rosilawati Rasli, Yoon Ling Cheong, M. Khairuddin Che Ibrahim, Siti Futri Farahininajua Fikri, Rusydi Najmuddin Norzali, Nur Ayuni Nazarudin, Nur Fadillah Hamdan, Khairul Asuad Muhamed, Afiq Ahnaf Hafisool, Ruziyatul Aznieda Azmi, Harith Aswad Ismail, Roziah Ali, Nurulhusna Ab Hamid, Mohd Zainuldin Taib, Topek Omar, Nazni Wasi Ahmad, Han Lim Lee

**Affiliations:** 1 Medical Entomology Unit, Infectious Disease Research Centre, Institute for Medical Research, National Institute of Health, Ministry of Health, Selangor, Malaysia; 2 Biomedical Epidemiology Unit, Special Resource Centre, Institute for Medical Research, Ministry of Health, Kuala Lumpur, Malaysia; 3 Biomedical Research, Strategic & Innovation Management Unit, Institute for Medical Research, National Institute of Health, Ministry of Health, Selangor, Malaysia; 4 Biomedical Museum Unit, Special Resource Centre, Institute for Medical Research, Ministry of Health, Kuala Lumpur, Malaysia; 5 Federal Territory Health Department of Kuala Lumpur and Putrajaya, Ministry of Health, Kuala Lumpur, Malaysia; Faculty of Science, Mahidol University, THAILAND

## Abstract

**Background:**

In Malaysia, dengue remains a top priority disease and usage of insecticides is the main method for dengue vector control. Limited baseline insecticide resistance data in dengue hotspots has prompted us to conduct this study. The present study reports the use of a map on the insecticide susceptibility status of *Aedes aegypti* and *Aedes albopictus* to provide a quick visualization and overview of the distribution of insecticide resistance.

**Method and results:**

The insecticide resistance status of *Aedes* populations collected from 24 dengue hotspot areas from the period of December 2018 until June 2019 was proactively monitored using the World Health Organization standard protocol for adult and larval susceptibility testing was conducted, together with elucidation of the mechanisms involved in observed resistance. For resistance monitoring, susceptibility to three adulticides (permethrin, deltamethrin, and malathion) was tested, as well as susceptibility to the larvicide, temephos. Data showed significant resistance to both deltamethrin and permethrin (pyrethroid insecticides), and to malathion (organophosphate insecticide) in all sampled *Aedes aegypti* populations, while variable resistance patterns were found in the sampled *Aedes albopictus* populations. Temephos resistance was observed when larvae were tested using the diagnostic dosage of 0.012mg/L but not at the operational dosage of 1mg/L for both species.

**Conclusion:**

The present study highlights evidence of a potential threat to the effectiveness of insecticides currently used in dengue vector control, and the urgent requirement for insecticide resistance management to be integrated into the National Dengue Control Program.

## Author summary

Insecticide resistance may threaten the efficacy of insecticides currently used in the dengue vector control program. These current findings reveal widespread insecticide resistance in *Aedes aegypti* and *Aedes albopictus* sampled from dengue hotspots. This screening provides comprehensive data on insecticide resistance to insecticides commonly used in dengue vector control programs. The mapping approach utilized in this present study facilitates easy visualization of the resistance distribution in dengue vectors across many dengue hotspots. Therefore, it could hence be beneficial to the Ministry of Health in managing the threat of resistance before it compromises current dengue vector control strategies.

## Introduction

Dengue is primarily caused by dengue virus (DENV) transmitted by *Aedes* species. This disease was recognized to be the fastest spreading vector-borne viral disease globally and is endemic in over 100 countries [1]. Hitherto, Malaysia has been significantly affected by dengue. The average number of dengue cases and deaths were 123,839 cases and 222 deaths respectively per annum, over the periods from 2014–2018. In 2019, an increase of 62% infections and a surge of 22% deaths were reported, compared to the previous year. During 2019, Selangor continued to see the highest number of dengue cases in Malaysia with more than 72,000 cases which was 55% of all cases reported in Malaysia [2,3]

With no medical cure for dengue currently available [4,5]. Mosquito vector control remains one of the best tools for dengue prevention. Insecticide-based control plays a major role among other dengue control activities [4]. Therefore, resistance to these insecticides in mosquito vector populations may threaten the efficiency of current dengue control programs.

In order to implement insecticide resistance management strategies, the exploration of underlying mechanisms is required to provide key data for elucidating the underlying causes of resistance and, thus, aid in the development of appropriate counter measures to control the spread of resistance in *Aedes* vector populations. In general, there are two common pathways associated with resistance development: detoxification of insecticides via metabolic enzymes [6–8] and mutations in insecticide target site receptors [9–12]. Most studies reporting resistance in *Aedes* spp. focus on pyrethroids and relatively little is known about organophosphate resistance mechanisms associated with the target site acetylcholinesterase (AChE). This is probably due to pyrethroids being more heavily used than organophosphates in public health vector control programs. In Malaysia, pyrethroids are the main adulticides used to control the adult stage, whilst temephos is used as a larvicide [13]. Several studies have previously reported the occurrence of pyrethroid resistance in *Aedes spp*. in Malaysia [14–20]. However, there have been no previous reports of *Aedes spp*. resistant to temephos at the operational dosage in Malaysia.

Presently, there is a limitation on the centralized and systematic collection of insecticide resistance data, especially from dengue hotspots. Indeed, this information is critically needed as baseline data to tackle the threat of possible exponential increase in insecticide resistance in dengue vector populations in Malaysia. This present study provides up to date information on dengue vector resistance profiles with respect to *Aedes* spp. as well as elucidating the mechanisms of resistance. These data are reviewed in the light of background data on resistance from recently published papers. These valuable resources are important for designing an appropriate resistance management strategy for *Aedes sp*. particularly in dengue hotspots in Malaysia.

## Methods

### Study site

Mosquito sampling was conducted in dengue hotspots in Selangor state. A collaborative partnership with Selangor Health District Officer to engage site selection for mosquito sampling was established. A total of 24 pre-selected study sites were used for the purpose of mapping insecticide resistance status of dengue vector, with the criteria for pre-selection being:—(1) residential area with continuously reported dengue cases > 32 days; (2) Intensive use of chemical insecticide control activity; and (3) high *Aedes* density.

### Mosquito collection

In total, 1440 ovitraps were deployed to complete mosquito collection in the 24 hotspots. A total of 60 ovitraps were deployed at each site, where ovitraps was placed inside (n = 30) and outside (n = 30) of 30 randomly selected houses. The ovitrap is a 300mL black plastic container with measurement of 7.8cm diameter opening, 6.5cm base, and 9.0cm height, containing water that was added to a level of 5.5cm together with a wood paddle (10cmX2.5cmx0.3cm) for female to oviposit on. After 5 days of deployment in the field, all recovered ovitraps were collected and were brought back to the laboratory for species identification [21, 22]. The management of ovitraps (n = 1440) in field as well as data analysis in laboratory was managed via a mobile application, Pestrapp.

### Mosquito rearing

Identified larvae (F^0^) were then reared in the insectarium of the Medical Entomology Unit, Institute for Medical Research at 26 ± 2°C and 80% relative humidity [23]. The larvae were fed with liver powder once daily until they pupated. Pupae were collected and transferred to emergence bowls, which were placed into adult colony cages (30 x 30 x 30 cm). Once emerged, adult mosquitoes were provided with 10% sucrose solution ad libitum. Mosquitos aged 4 days were blood feed using mice to obtain F1 mosquitoes: the study protocol for feeding the mosquito was approved by the Ministry of Health’s Medical Research and Ethic Committee (MREC) has approved the study protocol (NMRR-18-12-39653). Three days after blood feeding, a cylindrical black plastic cup filled with one third volume of water and lined with filter paper for oviposition was placed into the adult cage. The adult mosquitoes were allowed to oviposit for 3 days duration. The eggs of F1 mosquito obtained were used for experiments.

### Insecticide susceptibility testing

This study employed the gold standard protocol of insecticides susceptibility adult and larval assays described by the WHO (2016) [24]. A laboratory insecticide susceptible strain of *Aedes aegypti* (F1074) was used as reference strain, whilst collected field populations of *Aedes aegypti* (F1) were used as resistance monitoring samples.

For adult susceptibility assays, unfed female mosquitoes aged 3 to 7 days old were used. Treatment and control assays each consisted of 4 replicates of test mosquitoes (n = 100) exposed to tested insecticides paper and 2 replicates of test mosquitoes (n = 50) exposed to control paper. All tested mosquitoes were exposed to test insecticides for 1 hour. Test mosquitoes were held for 24-hours of recovery period at optimum conditions with 27 ± 2°C temperature and 75 ± 10% relative humidity and were given with 10% sugar solution, before mortality rate was recorded. For larva assays, 4 replicates of test larvae at late 3rd instar stage (n = 100) were introduced into treatment cups: each cup containing 600μl of 50mg/L temephos mixed with 249.4mL of absolute ethanol, and 2 replicates test larvae (n = 50) exposed to control cup containing 1mL of absolute ethanol mixed with 249mL of seasoned water. The larval mortality rate was observed at 24-hours post exposure. A table with discriminating concentrations and exposure time of insecticides used is provided as shown in Table 1.

**Table 1:** Discriminating concentration of tested insecticides and the diagnostic time of insecticide exposure.

Who susceptible test	Insecticide	Discriminating concentration	Exposure Time
Adult assay	Permethrin	0.25%	60 min
	Deltamethrin	0.03%	60 min
	Malathion	0.8%	60 min
Larval assay	Temephos	0.012mg/L	24 h
		1.0mg/L	24 h

### Phenotypic assessment of oxidase enzyme via synergist assay

We followed the synergist-insecticide bioassay protocol described by the WHO [25]. The transfer techniques of mosquito, number of mosquitos used, as well as interpretation of 24-h mortality is similar to the standard WHO adult susceptibility assay. Four sets of assays were conducted as follows: (set 1) test mosquitoes were exposed to PBO only, (set 2) test mosquitoes were exposed to PBO then to deltamethrin, (set 3) test mosquitoes were exposed to deltamethrin only, (set 4) mosquitoes were exposed to control paper only. Exposure to each paper was 1 hour. Later, test mosquitoes were held for 24-hours of recovery period before the mortality rate was recorded.

### Elucidation of underlying resistance mechanism via biochemical enzyme assays

#### Mixed function oxidase enzyme assay

The protocol described by Brogdon [26] and Nazni et al with a slight modification [27] were employed. Firstly, 500μL of 0.25M sodium acetate buffer (pH 5.0) was added into a 1.5mL microcentrifuge tube containing an individual mosquito and the mosquito homogenized. The homogenate was centrifuged at 15,000 rpm at 4°C for 5 minutes. Then, four replicates of 100μL individual mosquito homogenate were aliquoted into a 96-well microplate. A total of 200μL of 0.0021M 3,3’,5,5’-tetramethylbenzidine (TMBZ) was added into each well containing the homogenate as a substrate solution and incubated for 1 min. Then, 25μL of 3% hydrogen peroxide solution was added into each well to stop the reaction. The color changes took place immediately from blue to colorless. The reaction was allowed to continue at room temperature for 10 min. The optical density (OD) was then determined at 630nm using an immunoassay reader.

#### Esterase enzyme assay

The protocol described by Brogdon [28] and Lee [29] were employed. Firstly, 500μL of the 2.0M phosphate buffer solution (PBS, pH 7.6) was pipetted into a 1.5mL microcentrifuge tube containing an individual mosquito which was then homogenized. The homogenate was centrifuged at 15,000 rpm at 4°C for 5 minutes. Then, four replicates of 100μL individual mosquito homogenate were aliquoted into a 96-well microplate. A total of 100μL of 0.0322M naphthyl acetate was added into each well and incubated for 1 min at room temperature (28°C). Thereafter, 100μL of Fast Blue stain was added into each well and incubated for 10 min at room temperature (28°C). Change of color took place immediately from clear to pink purple, and then to blue following the incubation period. Then, a total of 50μL of 10% acetic acid was added into each well to stop the reaction. The absorbance of the enzymatic reaction was measured after 10 min at 450nm.

#### Insensitive acetylcholinesterase (AChE) assay

Modification of Ellman’s method described by Brogdon [30] and the protocol of Scott and McAllister [31]. Firstly, 500 μL of 0.25M sodium acetate buffer (pH 5.0) was added into a 1.5mL Eppendorf tube containing an individual mosquito which was homogenized. Then, the homogenate was centrifuged at 15 000 rpm at 4°C for 5 min. Eight replicates of 50μL individual mosquito homogenate were aliquoted into a 96-well microplate. A volume of 50μL of 10% acetone buffer solution of acetylthiocholine iodide (ASCHI) plus 2mg/ L of propoxur was added into 4 replicates and 50μL of 0.0026M ASCHI solution without propoxur was added into another 4 replicates. Thereafter, 50μL aliquot of dithiobis 2-nitrobenzoic acid (DTNB) was added into each well. A colour reaction took place immediately to yellowish colour or colourless. A faint colorless solution would indicate the presence of sensitive AChE in the replicates inhibited by propoxur, while an intense yellowish solution would indicate the presence of insensitive AChE as the enzyme is not be inhibited by propoxur. The OD value of all reactions was measured at 410nm after 30 min of incubation at room temperature.

### Mapping of insecticide susceptibility data

Insecticide susceptibility level and metabolic enzyme resistance of both *Aedes aegypti* and *Aedes albopictus* were mapped using QGIS software version 3.8 [32]. This involves a total of 180 insecticide bioassay data of 24-h mortality rate and enzyme activities of each sampled *Aedes* mosquito population. The underlying road network in gray is adapted from “Malaysia Roads” of MapCruzin.com; the water and forest area, that are indicated in blue and green are derived from OpenStreetMap.org under the Open Database License [https://opendatacommons.org/licenses/odbl/1.0/].

## Results

### Insecticide susceptibility status of *Aedes sp*. against adulticides deltamethrin, permethrin, malathion and temephos

**Fig 1:**
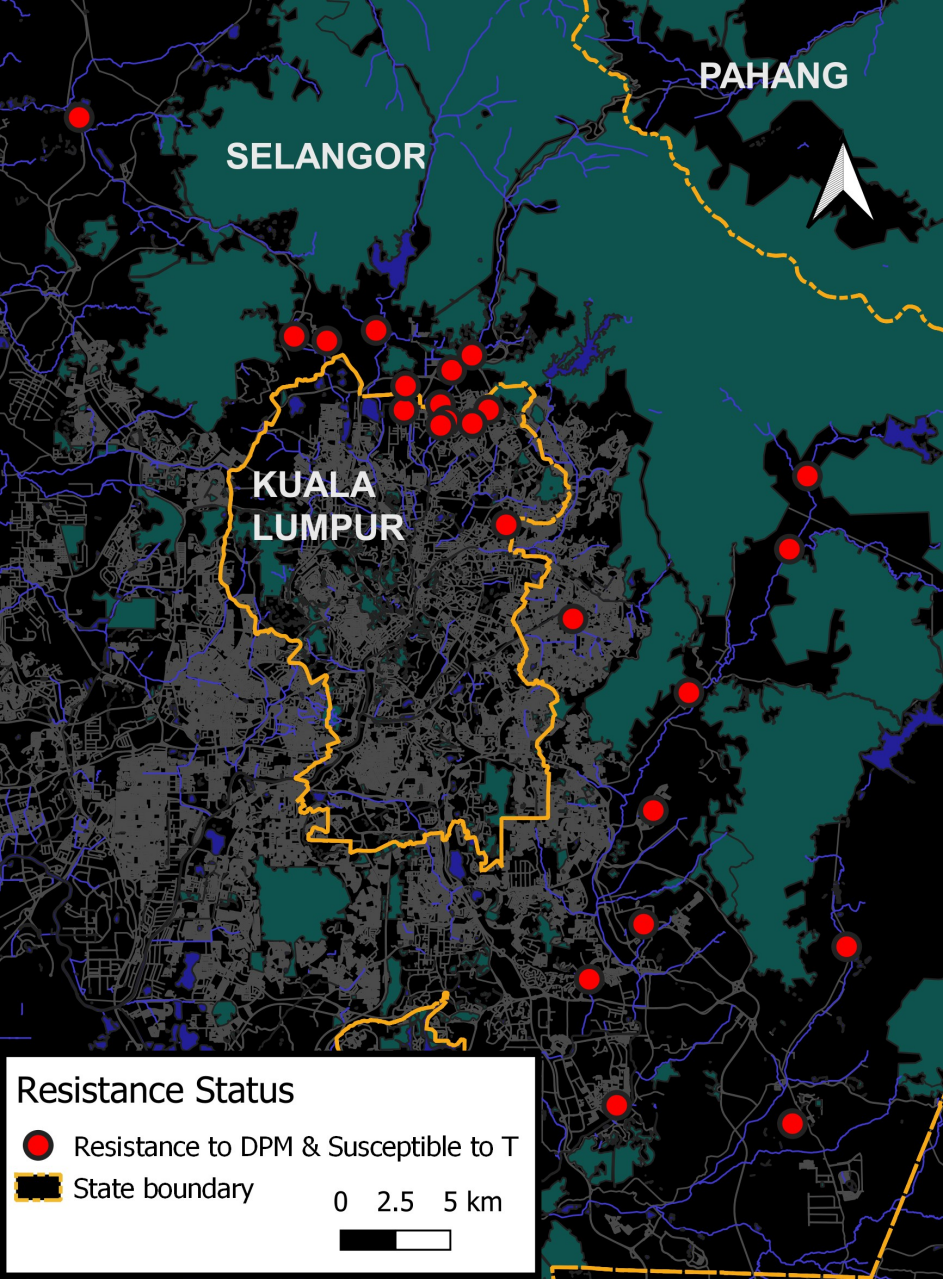
Insecticide susceptibility profile of *Aedes aegypti* populations. The code D,P,M, and T refers to deltamethrin, permethrin, malathion, and temephos, respectively.

**Fig 2:**
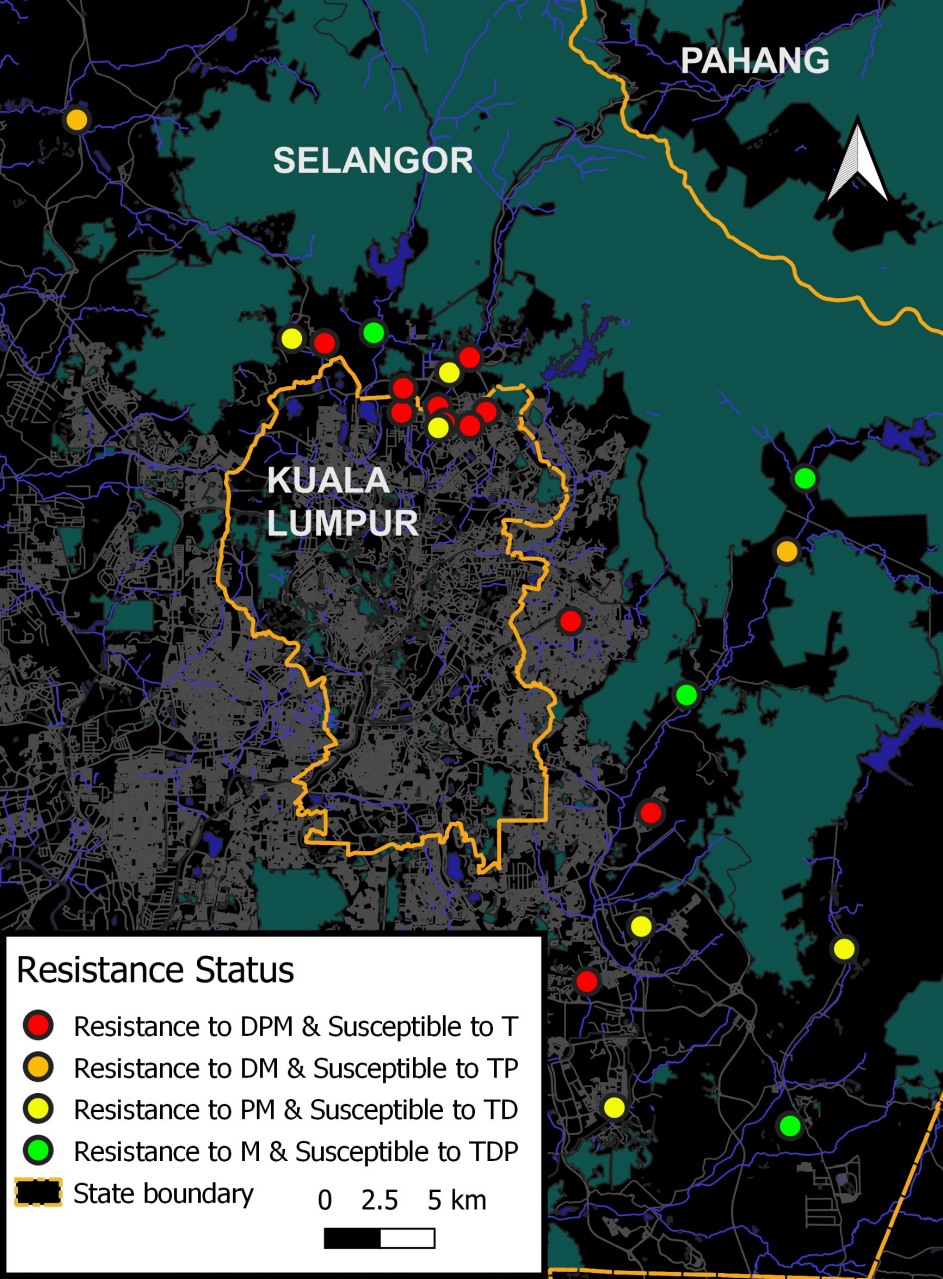
Insecticide susceptibility profile of *Aedes albopictus* populations. The code D, P, M, and T refers to deltamethrin, permethrin, malathion, and temephos, respectively.

Result interpretation of 24-h adult assays adhered to the recommendation by the WHO, 2016 [24]. Susceptibility is indicated when mortality is more than 98% to 100%, while resistance is indicated when mortality was less than 98%. The maps assist in visualizing resistance profiles between *Aedes* species. In overall view, maps as shown in Figs 1 and 2 clearly displayed that *Aedes aegypti* populations were more resistant than *Aedes albopictus* populations against all tested adulticides.

Almost all populations of *Aedes aegypti* displayed a similar pattern of multiple resistance which was marked with 24 red dots (Fig 1); indicating resistance to OP (malathion) and PYs (deltamethrin and permethrin). On the other hand, all *Aedes albopictus* populations were found to be resistant to malathion, and 11 *Aedes albopictus* populations were multi-resistant (S1, S3, S5, S6, S7, S8, S11, S15, S19, S23 and S24) being resistant also to PYs (deltamethrin and permethrin); this was marked with red dots in Fig 2. In addition, 8 *Aedes albopictus* populations (S2, S4, S10, S12, S14, S20, S21 and S22) were resistant to OP (malathion) and resistant to one of the PY insecticides (permethrin or deltamethrin)—denoted with yellow and orange dots in map of Fig 2- this demonstrated multiple resistance and cross resistance development. Despite resistance to malathion being found in all populations, only 4 *Aedes albopictus* populations (S9, S16, S17 and S18) were susceptible to PYs (deltamethrin and permethrin); denoted with green dots as shown in map of Fig 2.

Overall screening of the insecticide susceptibility status of *Aedes* mosquitoes from dengue hotspots showed evidence of high levels of resistance against the tested adulticides deltamethrin, permethrin and malathion.

### Susceptibility status of *Aedes sp*. against larvicides temephos

Overall, the larvae of all *Aedes* populations showed a mortality of <90% at a temephos diagnostic dosage of 0.012mg/L, indicating these populations were resistant to temephos at the diagnostic dosage (See S1 Table). Nevertheless, temephos at 1 mg/L is still effective in causing complete larval mortality in *Aedes aegypti* and *Aedes albopictus* populations at this operational application dosage (Figs 1 and 2).

#### Impact of biochemical metabolic enzyme

**Fig 3:**
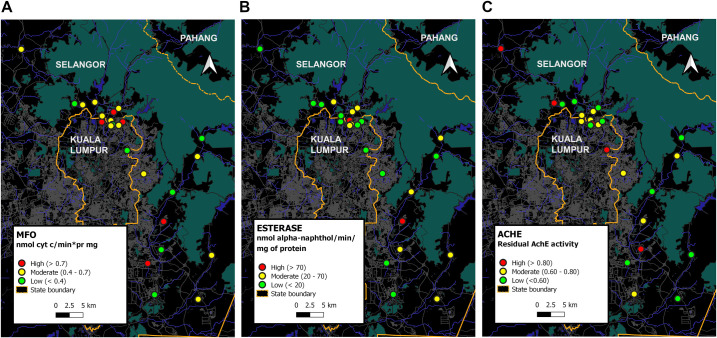
Enzyme Mix function oxidase (MFO), esterase and residual acetylcholinesterase (AChE) activity in collected *Aedes aegypti* populations.

**Fig 4:**
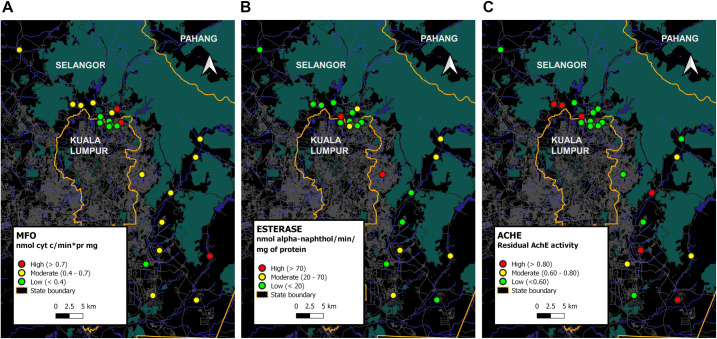
Enzyme Mix function oxidase (MFO), esterase and residual acetylcholinesterase (AChE) activity in collected *Aedes albopictus* populations.

A great difference in metabolic enzyme activities was discovered among *Aedes* mosquito populations. In general, all three enzyme activities of MFO, esterase and insensitive AChE were grouped into high, moderate and low (Figs 3 and 4); MFO activity (cytochrome C/min/mg/protein mg) was >0.7, 0.4 to 0.7, and 0.1 to 0.3, while esterase activity (nmol α-naphtol/min/protein mg) was >70, 20 to 70, and <20; AChE activity was >80%, and 60% to 80%, and <60%. The maps in Figs 3 and 4 displays the variation in resistance mechanisms across geographical areas and across species collected from the same collection site.

Figs 3 and 4 revealed a diversity of enzymatic profiles for in both species. Firstly, high/moderate MFO, high/moderate esterase, and insensitive AChE activity in 3 *Aedes aegypti* populations (S4, S6, S19) and 2 *Aedes albopictus* populations (S21, S22). Secondly, high/moderate MFO, low esterase and insensitive AChE activity in 7 *Aedes aegypti* populations (S2, S5, S10, S11, S15, S22, S23) and 5 *Aedes albopictus* populations (S1, S14, S16, S17, S18). Thirdly, high/moderate MFO, low esterase and insensitive AChE activity in 3 *Aedes aegypti* populations (S1, S3, S24) and 6 *Aedes albopictus* populations (S2, S3, S4, S9, S12, S19). Fourthly, high/moderate MFO, moderate esterase, and insensitive AChE activity in 6 *Aedes aegypti* populations (S8, S9, S10, S12, S14, S16) and 3 *Aedes albopictus* populations (S8, S20, S23). Sixthly, low MFO, moderate esterase, insensitive AChE enzyme in 2 *Aedes aegypti* populations (S17, S7) and 1 *Aedes albopictus* populations (S11). Seventhly, low MFO, moderate esterase, sensitive AChE enzyme in 3 *Aedes aegypti* populations (S20, S21, S18) and 1 *Aedes albopictus* populations (S10). Eighthly, low MFO, low esterase and insensitive AChE activity in 3 populations of *Aedes aegypti* (S12, S13, S14) and 3 *Aedes albopictus* populations (S7, S15, S24). Finally, low MFO, low esterase and sensitive AChE was seen in 2 *Aedes albopictus* populations (S5, S6).

#### Resistance mechanisms-associated- pyrethroids resistance

The effect of the synergist PBO enhanced the toxicity of deltamethrin in all collected *Aedes aegypti* populations but with just one population (S19) reaching 100% mortality. For *Aedes albopictus* populations, there was a significant enhancement with complete mortality observed in only 13% (n = 3) of resistant *Aedes albopictus* populations (S5, S12 and S14); while a slight increase in toxicity was observed in remaining 65% (S1,S2,S4,S17, S19, S3, S6, S7, S15, S24, S10, S11, S8 S21 and S22) resistant *Aedes albopictus* populations except in 2 populations (S22 and S19).

#### Resistance mechanisms-associated- organophosphate resistance

Result showed that insensitive AChE and esterase enzyme can evidently involve in development of resistance to malathion in *Aedes* mosquito. Estimation of allelic frequency associated with ACE gene mutation based on residual activity of AChE signified resistant homozygous population (>0.8nm); heterozygous population (0.6nm– 0.8nm), and susceptible homozygous population(<0.5nm) (Figs 3C and 4C)

For *Aedes aegypti* populations, 22% (n = S2, S7, S13, S14, and S17) were resistant homozygous individuals; 35% (n = S4, S5, S6, S11, S12, S15, S19, and S23) were heterozygous individuals, and 43% (n = S1, S3, S8, S9, S10, S16, S18, S20, S21, and S24) were susceptible homozygous (Fig 3C). On the other hand, of all *Aedes albopictus* populations tested, 26% (S1, S11, S14, S16, S17 and S18) were resistant homozygous individuals; 22% (S7, S15, S21, S22, S24) were heterozygous individuals, and 52% (S2, S3, S4, S5, S6, S8, S9, S10, S12, S19, S20 and S23) susceptible homozygous individuals (Fig 4C).

## Discussion

Over half million people across the globe are at risk of dengue viral infection. This disease is spread mainly by infected *Aedes* mosquitoes (Culicidae, Diptera) [30]. The growing demand on effective tetravalent vaccine for dengue preventive interventions means that has limitedly availability, which means that vector control is the key strategy to control transmission of dengue virus [4]. Unfortunately, the approach of chemical insecticide-based is facing challenges due to rising insecticide resistance in mosquito vector populations.

The question to what extent does insecticide resistance impact the effectiveness of vector program is still contentious. Hence, the current study was conducted to update data on the insecticide susceptibility status in *Aedes* vector from dengue hotspots to common adulticides and larvicide used in vector control program in Malaysia. Results highlight a serious concern regarding the prevalence of resistant populations to deltamethrin, permethrin and malathion; *Aedes aegypti* population was attentively found to be more resistant to both pyrethroids tested than *Aedes albopictus* (Figs 1 and 2). The results obviously showed multiple resistances pattern in all *Aedes aegypti* populations. In the past 5 years, 3 publications have reported widespread resistant *Aedes aegypti* to pyrethroids and mostly susceptible to malathion [17–19]. For current monitoring, resistance to malathion detected in not only *Aedes aegypti* but as well in all populations of *Aedes albopictus*, thus conversely limit the use of adulticides.

There are four possible explanation may be attributed for current reporting outcomes of resistance to malathion. Firstly, intense exposure to malathion if this chemical is used heavily to curb dengue cases dengue hotspots. Secondly, current diagnostic dosage in latest guidelines is 0.8%, whereas previously for malathion diagnostic dosage was 5% for *Anopheles sp*., and this was adapted for *Aedes* testing. Here, increment of 84% in active ingredient than current diagnostic dosage is seemingly limitless to trace malathion resistance development in *Aedes* vectors. Thirdly, high frequency of resistant homozygous individuals with dominant and heterozygous genotype of resistance ACE-1 in population. Fourthly, impact by malathion usage in agriculture sector particularly *Aedes albopictus* in geographical sub-urban setting near to agriculture land.

In authors’ opinion, evaluation of frequency ACE-1 allelic provides concrete evidence-based data revealing role of ACE-1 gene mutation in the development of malathion resistant population observed in almost half of tested *Aedes* populations, and correspond to current assessment of high frequency of homozygous and heterozygous resistant population individuals detected in 52% (n = 12,sites) and 43% (n = 10, sites) in *Aedes albopictus* populations and *Aedes aegypti* populations, respectively (Figs 3C and 4C). Consequently, recovery of malathion resistance is a tremendous challenge in locality with *Aedes* population that were not only resistant to malathion but resistant as well to pyrethroids insecticides. In another view point to dismiss uncertainty, malathion was no longer used as adulticide for dengue control since 1999. Further exploration on insecticide-based pest control in agriculture is warranted to evidently explain and confirm if resistance development in dengue vector may be impacted by this factor as well.

We have reported on the basis of previous findings on pyrethroid resistance in local *Aedes* species collected from dengue hotspots, with the finding’s discoveries that distribution of resistance to pyrethroid has been widespread, and is localized; wherein some local populations were characterized by cross-resistance to pyrethroids thus creating a patchy mosaic pattern. The adaptation of mosquito which lead to resistance development to pyrethroid is likely attributable to intense exposure to insecticides(s) and selection pressure, hence this is expected to happen in most hotspots area, where dengue cases were continuously reported and control of dengue prevention is highly dependent on targeted spraying of pyrethroid insecticides to reduce vector density inside and within 400 meters radius surrounding index houses of reported patients.

In the study, the present result showed PBO is limited in its effectiveness to overcome multiple resistant *Aedes* mosquito characterized by multiple enzyme resistance, with evidence that almost all resistant *Aedes aegypti* and *Aedes albopictus* were not fully impacted by deltamethrin along with PBO (<98% mortality), except for only 1 *Aedes aegypti* population collected from S19, and 3 *Aedes albopictus* populations collected from S5, S12,and S14 (Figs 3A and 4A). This, indicated not only enzyme of oxidases, but esterase detoxification could well be involved in implication of pyrethroids toxicity as observed in *Aedes albopictus* populations collected from S8, S21 and S22, and in *Aedes aegypti* populations collected from S4, S6, S7, S8, S9, S10, S16, S17, S18, S20 and S21 (Figs 3B and 4B). From PBO synergist assays, screening of resistant *Aedes* mosquitoes populations characterised with (1) low MFO and low esterase enzymes as observed in *Aedes albopictus* populations collected from S5, S6, S7, S15 and S24 and in *Aedes aegypti* populations collected from S12, S13 and S14; and those populations characterised with (2) high or moderate MFO enzyme activity with less or no impact of PBO along with insecticide pyrethroids, should further identify the role of *kdr* gene as a contributing factor for the development of insecticide resistance.

In the event where metabolic enzyme is the cause of resistance, addition of synergist, such as piperonyl butoxide (PBO) was reported to enhance the insecticidal effect of pyrethroid insecticide [15]. However, looking at the susceptibility data of current study, PBO which reacts via inhibiting MFO enzyme are no longer useful in reverting the resistance occurrence in most populations, indicating no significance role of oxidase in permethrin resistance. Nevertheless, we recommend the conduct of PBO synergist assay in a laboratory lacking molecular facilities and expertise in molecular field to pre-determined involvement of *kdr* gene in permethrin resistance.

For temephos susceptibility status, previous reports of temephos susceptibility status [33] are consistent with our latest screening of *Aedes* population against temephos. In spite of the fact that temephos resistance at diagnostic dosage was widespread, it was noted that *Aedes* mosquito populations were still susceptible to temephos at operational dosage of 1.0mg/L; meaning, temephos usage in vector control program is still effective without control failure (Figs 1 and 2 and S1 Table). From larval assay findings, it was noteworthy showed that reporting resistance data in *Aedes* vector using WHO diagnostic dosage provides early warning of resistance, and does not imply immediate control failure because these populations were still susceptible to application dosage. As battling dengue is highly dependent on vector control interventions, it is important to have timely information on resistance occurrence and severity. With the susceptibility profiles data, remedial measures should be taken early to avert control failure, and most importantly, to preserve the usefulness vector control products.

Given the current IR reports, strategies involving insecticide rotation; combining intervention of adult and larval control simultaneously, and mosaic strategy—where a compound is used in one geographical area while different insecticide classes and mode of action is used in neighboring areas—is warranted to overcome cross-resistant population. Further challenges dealing with multiple resistant populations has restricted the choice of insecticides that can be used in adulticiding interventions, hence innovative non-insecticidal control tool such as release of *Wolbachia* infected mosquito [34], sterile insect technique (SIT) or genetically modified mosquito [35,36] are urgently needed to effectively control disease transmission.

The effort of managing insecticide resistance is key to ensuring the success of chemical insecticide interventions in control program are effective [37]. Although some studies on insecticide resistance of dengue vectors were conducted in Malaysia, there is still a paucity of data available. Most reported studies are focused on reporting susceptibility level with absence of complementary resistance mechanisms data. In addition, among all IR *Aedes* vector studies conducted so far in Malaysia, there are few reports on the susceptibility status of *Aedes* vector populations collected particularly from dengue hotspot sites. Challenges to managing resistant dengue vectors are alarming when considering the increased number of hotspots reported, especially in Klang Valley. This report provides comprehensive resistance data on local *Ae*. *aegypti* and *Ae*. *albopictus* species from dengue hotspot areas and is the most extensive in the of the number of sampling sites to date. This provides the latest baseline insecticide susceptibility data and suggests the necessity for more extensive insecticide resistance monitoring in *Aedes* vectors from all reported dengue hotspots. This will inform the rolling out of a systematic insecticide resistance management strategy in Malaysia.

In order to plan an effective insecticide resistance management program, a call to strengthen the capacities of operational teams and sentinel laboratories to conduct routine insecticide monitoring to screen the susceptibility levels and elucidate resistance mechanisms are crucially important, as timely detection of resistance allows visibility on changes in resistance over time. Despite efforts to monitor resistance development in *Aedes* vectors, data pertaining to resistance mechanisms is crucial for the key decision makers on changes to insecticides and control practices. The impact of strategies to manage resistance should be evaluated to monitor reversion of resistant population to susceptibility. In reality, vector control departments with the complexities of a multitasking environment face the challenge of having sufficient capacity to routinely conduct insecticide resistance testing on mosquito vector populations. The need to strengthen the capacities must be assessed. By continuing the current practice of using insecticides in most dengue hotspots without effective resistance monitoring will further extend the challenge of controlling resistant populations and, as a worst-case scenario, when application dosages are no longer effective in controlling mosquito vectors, this will compromise human protection to arthropod-borne disease in urban and sub-urban area using chemical is no longer promising, resulting in wastes of resources, in term of money, time and personnel. Therefore, fostering a strong relationship and collaboration between operational agencies, sentinel and core laboratories, and academic research to link the input of resistance data including resistance mechanisms of *Aedes* mosquito in a centralized systematic data management system is crucial.

## Supporting information

S1 Table Insecticide susceptible larva assay data of *Aedes aegypti* and *Aedes albopitus* against temephos at diagnostic and operational dosage of 0.012mg/L and 1.0mg/L, respectively. (DOCX)
