# Supplementary material for: Insecticide resistance in dengue vectors from hotspots in Selangor, Malaysia
Source: PLoS Negl Trop Dis. 2021 Mar 23;15(3):e0009205. doi: 10.1371/journal.pntd.0009205 (PMC7987141; doi:10.1371/journal.pntd.0009205)
Supplement: S1 Table — (DOCX) [file pntd.0009205.s001.docx]

**S1 Table. Larval mortality of *Aedes sp.* at 24 hours post-exposure to temephos at diagnostic concentration and operational dosage of 0.012mg/L and 1.0mg/L, respectively**. Highlighted cell indicating confirmed resistance; ** symbol indicating susceptible populations, SD indicating standard deviation, NA indicating not available, as there was absence of respective *Aedes* sp. collected from the site.

| **Sites** | **Insecticide** | **Temephos 0.012** | | | | **Temephos 1.0mg/L** | | | |
| --- | --- | --- | --- | --- | --- | --- | --- | --- | --- |
|  | **Strain** | ***Aedes aegypti*** | | ***Aedes albopictus*** | | ***Aedes aegypti*** | | ***Aedes albopictus*** | |
|  | **Site coordinate** | **%**  **mortality** | **SD** | **%**  **mortality** | **SD** | **%**  **mortality** | **SD** | **%**  **mortality** | **SD** |
| S1 | 3.250643, 101.658943 | 0% | 0.00 | 0% | 0.00 | 100%** | 0.00 | 100%** | 0.00 |
| S2 | 3.342326, 101.557382 | 3% | 0.04 | 7% | 0.02 | 100%** | 0.00 | 100%** | 0.00 |
| S3 | 3.222379, 101.725211 | 0% | 0.00 | 0% | 0.00 | 100%** | 0.00 | 100%** | 0.00 |
| S4 | 3.238618, 101.710116 | 3% | 0.04 | 1% | 0.02 | 100%** | 0.00 | 100%** | 0.00 |
| S5 | 3.222220, 101.690543 | 3% | 0.02 | 0% | 0.00 | 100%** | 0.00 | 100%** | 0.00 |
| S6 | 3.224486, 101.705516 | 16% | 0.17 | 11% | 0.06 | 100%** | 0.00 | 100%** | 0.00 |
| S7 | 3.217997, 101.708158 | 7% | 0.05 | 2% | 0.02 | 100%** | 0.00 | 100%** | 0.00 |
| S8 | 3.244780, 101.718431 | 6% | 0.08 | 17% | 0.09 | 100%** | 0.00 | 100%** | 0.00 |
| S9 | 3.254962, 101.679125 | 10% | 0.05 | 100%** | 0.00 | 100%** | 0.00 | 100%** | 0.00 |
| S10 | 3.215958, 101.705612 | 0% | 0.00 | 3% | 0.04 | 100%** | 0.00 | 100%** | 0.00 |
| S11 | 3.232171, 101.691210 | 19% | 0.12 | 25% | 0.09 | 100%** | 0.00 | 100%** | 0.00 |
| S12 | 2.937040, 101.777803 | 21% | 0.08 | 14% | 0.08 | 100%** | 0.00 | 100%** | 0.00 |
| S13 | 3.175208, 101.732465 | 0% | 0.00 | NA | NA | 100%** | 0.00 | NA | NA |
| S14 | 3.252513, 101.645535 | 0% | 0.00 | 0% | 0.00 | 100%** | 0.00 | 100%** | 0.00 |
| S15 | 3.216763, 101.718553 | 1% | 0.02 | 0% | 0.00 | 100%** | 0.00 | 100%** | 0.00 |
| S16 | 2.929523, 101.849863 | 74% | 0.27 | 78% | 0.26 | 100%** | 0.00 | 100%** | 0.00 |
| S17 | 3.011356, 101.788825 | 4% | 0.06 | 4% | 0.06 | 100%** | 0.00 | 100%** | 0.00 |
| S18 | 3.106338, 101.807370 | 5% | 0.04 | 8% | 0.07 | 100%** | 0.00 | 100%** | 0.00 |
| S19 | 3.057969, 101.792785 | 0% | 0.00 | 4% | 0.00 | 100%** | 0.00 | 100%** | 0.00 |
| S20 | 3.195198, 101.855959 | 17% | 0.09 | 24% | 0.03 | 100%** | 0.00 | 100%** | 0.00 |
| S21 | 3.002108, 101.872046 | 73% | 0.40 | 84% | 0.19 | 100%** | 0.00 | 100%** | 0.00 |
| S22 | 3.165214, 101.848585 | NA | NA | 3% | 0.04 | NA | NA | 100%** | 0.00 |
| S23 | 3.136611, 101.759865 | 1% | 0.02 | 4% | 0.03 | 100%** | 0.00 | 100%** | 0.00 |
| S24 | 2.988740, 101.766514 | 3% | 0.04 | 3% | 0.04 | 100%** | 0.00 | 100%** | 0.00 |
